# The Profile and Treatment Outcomes of the Older **(**Aged 60 Years and Above**)** Tuberculosis Patients in Tamilnadu, South India

**DOI:** 10.1371/journal.pone.0067288

**Published:** 2013-07-08

**Authors:** Ramya Ananthakrishnan, Kaliyaperumal Kumar, Marimuthu Ganesh, Ajay M. V. Kumar, Nalini Krishnan, Sowmya Swaminathan, Mary Edginton, Arunagiri K, Devesh Gupta

**Affiliations:** 1 REACH, Chennai, India; 2 International Union against TB and Lung disease (The Union), South East Asia Regional Office, New Delhi, India; 3 National Institute of Research on Tuberculosis, Chennai, India; 4 International Union against TB and Lung disease (The Union), Paris, France; 5 State Tuberculosis Office, Tamilnadu, India; 6 Central Tuberculosis Division, Ministry of Health and Family Welfare, Government of India, New Delhi, India; St. Petersburg Pasteur Institute, Russian Federation

## Abstract

**Background:**

With changing demographic patterns in the context of a high tuberculosis (TB) burden country, like India, there is very little information on the clinical and demographic factors associated with poor treatment outcome in the sub-group of older TB patients. The study aimed to assess the proportion of older TB patients (60 years of age and more), to compare the type of TB and treatment outcomes between older TB patients and other TB patients (less than 60 years of age) and to describe the demographic and clinical characteristics of older TB patients and assess any associations with TB treatment outcomes.

**Methods:**

A retrospective cohort study involving a review of records from April to June 2011 in the 12 selected districts of Tamilnadu, India. Demographic, clinical and WHO defined disease classifications and treatment outcomes of all TB patients aged 60 years and above were extracted from TB registers maintained routinely by Revised National TB Control Program (RNTCP).

**Results:**

Older TB patients accounted for 14% of all TB patients, of whom 47% were new sputum positive. They had 38% higher risk of unfavourable treatment outcomes as compared to all other TB patients (Relative risk (RR)-1.4, 95% CI 1.2–1.6). Among older TB patients, the risk for unfavourable treatment outcomes was higher for those aged 70 years and more (RR 1.5, 95% CI 1.2–1.9), males (RR 1.5, 95% CI 1.0–2.1), re-treatment patients (RR 2.5, 95% CI 1.9–3.2) and those who received community-based Direct Observed Treatment (RR 1.4, 95% CI 1.1–1.9).

**Conclusion:**

Treatment outcomes were poor in older TB patients warranting special attention to this group – including routine assessment and recording of co-morbidities, a dedicated recording, reporting and monitoring of outcomes for this age-group and collaboration with National programme of non-communicable diseases for comprehensive management of co-morbidities.

## Introduction

India is the highest tuberculosis (TB) burden country in the world accounting for nearly 25% of the global TB [1]. The Revised National Tuberculosis Control Programme (RNTCP), implemented throughout the country, has been successful in achieving the global targets of 70% case detection and 85% treatment success rates in new smear positive TB patients since 2007(2). Over the next 5 years (2012–17), the program aims to achieve universal access to TB services with a treatment success rate of at-least 90% [2]. To achieve this, it is important to examine specific clinical and demographic subgroups who might be at high risk of poor treatment outcomes and to provide special attention to them if needed. Previous studies done in India, Hong-King, Ethiopia have shown that TB patients aged 60 years and above are one such sub-group with poor treatment success owing to poor treatment adherence, poor tolerance to therapy, higher mortality rates and co-morbidities [3]–[7].

India is undergoing a demographic transition and with increase in average life expectancy, the number of people who are 60 years and more (the defined age for senior citizen according to Indian legislation) is increasing [8]. Not only can we expect more older TB patients, but they could also present problems in clinical presentation, spectrum of disease and diagnosis for TB [9]–[12]. Persistence of TB in this age group may become an important source of infection and perpetuate the pool of infection in the community. Though previous studies in India between 1996 and 2005 have described poor treatment outcomes in this age group, these were conducted either in a tertiary care setting or were limited to a small geographical area [3], [5], [13]–[15]. Association of important factors including the type of TB, HIV status, type of DOT (Directly Observed Treatment) provider (a health care provider administering treatment and providing patient support) have not been studied in great detail in older TB patients. There is a crucial need to understand these factors comprehensively, along with other demographic and clinical characteristics and to measure their association with treatment outcomes in the older TB patients. Hence, the present study was undertaken with the following objectives.

For TB patients registered under the RNTCP in 12 selected districts of Tamilnadu

1. To assess the proportion of older TB patients (60 years of age and more).
2. To compare the type of TB and treatment outcomes between older TB patients and other TB patients (less than 60 years of age).
3. To describe the demographic and clinical characteristics of older TB patients and to assess if they are associated with TB treatment outcomes.

## Methods

### Ethics considerations

Since the study involved a review of existing records with no patient interaction, informed consent was considered unnecessary and a waiver of informed consent was received. Ethics approval for the study was obtained from the Ethics Advisory Group of the International Union Against TB and Lung Disease, Paris, France and from the REACH (Resource group for Education and Advocacy for Community Health) Independent Ethics Committee, India.

### Study design

This was a retrospective cohort study involving a review of records maintained routinely by RNTCP.

### Setting

Tamilnadu, a southern state of India consists of 32 districts and a population of 72 million. The RNTCP in Tamilnadu has been implemented throughout the state and services for diagnosis and treatment of TB are integrated within the general health system. There is a District TB Center (DTC) for each district which has six to ten Tuberculosis Units (TU), each of which serves approximately 500,000 population. A separate TB register is maintained in each TU. All patients are diagnosed and registered for treatment as per the RNTCP DOTS guidelines. Under RNTCP every patient registered in the program receive receives treatment under direct observation (DOT). Adherence to treatment is facilitated by Direct Observation of Treatment (DOT) with patients able to choose between health centers and community volunteers trained for the purpose based on convenience of location and timing. All registered TB patients are offered HIV counselling and testing and for those diagnosed as HIV positive, they are linked to HIV care and support including ART (Anti-Retroviral Therapy) and CPT (Co-trimaxozole Preventive Therapy).RNTCP follows WHO defined nomenclature for disease classifications and treatment outcome and the same have been used for this study [16].

The study was conducted in 12 selected districts of Tamilnadu which covers 31.8 million [17], selected purposively based on the area of work of the principal investigator.

### Study population and Study period

All TB patients registered under RNTCP in the selected districts, in the quarter April to June 2011 were included as study participants. The data was collected during the period July to September 2012.

### Data source, variables and instrument

For all TB patients, aggregate information on numbers, type of TB and treatment outcomes were extracted from the RNTCP quarterly reports. For older TB patients, we collected information on the following variables – age, sex, type of TB, category of treatment, HIV status, type of DOT provider and treatment outcomes.

### Data Analysis and statistics

Data was double entered into an EpiData database (Version 3.1, EpiData Association, Odense, Denmark) by two independent data entry operators. The two databases were compared for consistency and all inconsistencies were resolved by referring to the original data collection format. For the purpose of analysis, cured and treatment completed were grouped together as favourable outcome and those in categories died, loss-to-follow-up [19] (referred to as ‘default’ in standard WHO nomenclature), failure, transferred out were grouped as unfavourable outcome. Univariate analysis was done and appropriate proportions were calculated to describe the demographic and clinical profile of older TB patients. Age-specific case notification rates were calculated separately for older TB patients and the rest of them. Bivariate analysis was done to examine possible associations of demographic and clinical variables with treatment outcomes. Relative Risks (RR) and 95% confidence intervals (CI) were calculated. Chi square test was used to compare proportions and P value of <0.05 was considered as statistically significant. To assess the independent effects of each variable after adjusting for other variables, a multivariate analysis using log-binomial regression was done and adjusted relative risks were calculated using STATA 12.1. All the factors found to be significantly associated during bivariate analysis in our study and HIV status (an important confounder in previous studies) were included in the multivariate analysis.

## Results

Of 10,477 TB patients registered in the 12 selected districts of Tamilnadu, 1485 (14%) were older TB patients (**Table 1**)**.** The proportion of older TB patients was highest among those categorized as New Sputum Positive (NSP) followed by New Sputum Negative (NSN) and treatment after failure. The case notification among older TB patients was calculated to be 259 per 100,000 population as compared to the other TB patients where it was found to be 142 per 100,000 population.

**Table 1:** Proportion of Older TB patients among the cohort of TB patients registered under RNTCP in 12 districts of Tamilnadu, South India, April to June 2011.

Type of TB	All TB patients	Number of Older TB patients	Proportion of Older TB patients a
New Sputum Positive	3926	702	18
New Sputum Negative	2878	449	16
New Extra-pulmonary	2207	173	8
New Others	2	0	0
Treatment After Relapse	615	75	12
Treatment After Failure	76	10	13
Treatment After Loss to follow up	456	44	10
Retreatment Others	317	32	10
Total	10477	1485	14

a All percentages rounded off.

TB-Tuberculosis; RNTCP-Revised National TB Control Programme.

Nearly half (47%) of all older TB patients were new smear positive cases as compared with 36% in other TB patients (p value <0.001) ; while the proportion of new extra-pulmonary was 12% in the older group as compared to 23% among others (p value <0.001). The proportion of community-based DOT among older TB patients was 13% as compared to 22% among all others (p value <0.001). Details about demographic and clinical profile of older TB patients can be seen in **Table 2** **.** Among the 23 (1.5%) HIV-infected patients, 61% were on ART and 57% were on CPT.

**Table 2:** Demographic and Clinical Profile of Older TB patients registered under RNTCP in 12 districts of Tamilnadu, South India, April to June 2011.

Category	Sub-category	Number (%)
**Total**		1485 (100)
**Sex**	Male	1193 (80)
	Female	292 (20)
**Age groups** (**years**)	60 to 69	1130 (76)
	70 to 79	318 (22)
	80 to 89	35 (2)
	90 and more	2 (0)
**Category of treatment**	Category 1	1324 (89)
	Category 2	161 (11)
**Classification of TB**	Pulmonary	1305 (88)
	Extra-pulmonary	180 (12)
**HIV status**	Positive	23 (2)
	Negative	1458 (98)
	Unknown	4 (0)
**Sputum smear conversion at the end of intensive phase** *	Positive	25 (4)
	Negative	625 (89)
	Unknown	52 (7)
**Direct Observed Treatment**	Community based	193 (13)
	Health centre based	1292 (87)

* Among NSP (N = 702).

All percentages rounded off.

TB – Tuberculosis.

RNTCP- Revised National TB Control Program.

Four patients whose outcomes were not recorded were not included for the subsequent analysis. Comparison of treatment outcomes between older (N = 1481) and other (N =  8992) TB patients showed that older TB patients have a significantly higher risk (RR-1.4, 95% CI 1.2–1.6) of unfavourable outcomes (16% compared with 11% for all others). This association was stronger among new TB patients (RR 1.6, 95% CI 1.6–1.9) as compared to retreatment TB patients (RR 1.2, 95% CI 0.9–1.5).

Disaggregated data of treatment outcomes among older TB patients by demographic and clinical characteristics is shown in **Table 3** **.** Within the subgroups, mortality is higher among those aged 70 years and more (RR-2.0, 95% CI 1.4–2.8), re-treatment cases (RR-1.8, 95% CI 1.2–2.8) especially treatment after relapse and those who were HIV positive (RR-1.7, 95% CI 0.6–4.9). Loss to follow up is more common among re-treatment cases (RR-3.3, 95% CI 2.2–4.9) especially treatment after loss to follow up and those on community-based DOT (RR-2.0, 95% CI 1.3–3.1).

**Table 3:** Comparison of Demographic and Clinical characteristics and the treatment outcomes among older TB patients registered under RNTCP in 12 districts of Tamilnadu, South India, April to June 2011.

Category	Sub-category	Total N (%)	Cured N (%)	Treatment Completed N (%)	Loss to Follow-up N (%)	Died N (%)	Failure N (%)	Transfer out N (%)
Age groups	60 to 70 years	1126	521 (46)	450 (40)	68 (6)	72 (6)	9 (1)	6 (1)
	70 years and more	355	124 (34)	153 (44)	31 (9)	**45** (**13**)	1 (0)	1 (0)
Sex	Male	1189	540 (45)	448 (38)	87 (7)	99 (8)	10 (1)	5 (1)
	Female	292	105 (36)	155 (53)	12 (4)	18 (6)	0 (0)	2 (1)
Category of TB	Category 1	1322	579 (44)	565 (43)	71 (5)	96 (7)	7 (1)	4 (0)
	category 2	159	66 (41)	38 (24)	**28** (**18**)	**21** (**13**)	3 (2)	3 (2)
Classification of TB	Pulmonary	1303	644 (49)	447 (34)	95 (7)	101 (8)	10 (1)	6 (1)
	Extrapulmonary	178	1 (0)	156 (88)	4 (2)	16 (9)	0 (0)	1 (1)
Type of TB	New Sputum Positive	701	579 (83)	23 (3)	44 (6)	49 (7)	5 (1)	1 (0)
	New Sputum Negative	449	0 (0)	391 (87)	23 (5)	31 (8)	2 (0)	2 (0)
	New Extra-pulmonary	172	0 (0)	151 (88)	4 (2)	16 (9)	0 (0)	1 (1)
	Treatment After Relapse	75	47 (63)	5 (7)	7 (9)	**13** (**17**)	2 (3)	1 (1)
	Treatment After Failure	9	6 (67)	1 (11)	2 (22)	0 (0)	0 (0)	0 (0)
	Treatment After Loss to follow up	44	12 (27)	7 (16)	**17** (**39**)	5 (11)	1 (2)	2 (5)
	Category 2 Others	31	1 (3.2)	25 (80.6)	2 (6.5)	3 (9.7)	0 (0)	0 (0)
HIV status^a^	Positive	23	4 (17)	14 (61)	2 (9)	**3** (**13**)	0 (0)	0 (0)
	Negative	1455	641 (44)	589 (41)	95 (7)	113 (8)	10 (0)	7 (0)
Direct Observed Treatment	Community based	193	87 (45)	66 (34)	**23** (**12**)	17 (9)	0 (0)	0 (0)
	Health centre based	1288	558 (43)	537 (42)	76 (6)	100 (8)	10 (1)	7 (0)
Total		1481^b^	645 (43)	603 (40)	99 (7)	117 (8)	10 (1)	7 (1)

All percentages rounded off.

TB – Tuberculosis.

RNTCP- Revised National TB Control Program.

a HIV status unknown not included.

b Treatment outcome missing excluded.

The association of demographic and clinical characteristics with unfavourable treatment outcomes along with their respective relative risks are shown in **Table 4** **.** Among older TB patients, risk for unfavourable treatment outcome was higher for those aged 70 years and more (Unadjusted RR 1.6,CI 1.3 to 2.0), males (Unadjusted RR-1.5,CI 1.1 to 2.2), re-treatment patients (Unadjusted RR-2.6, CI 1.2 to 3.3) and those who received community-based DOT (Unadjusted RR-1.4 ,CI 1.0 to 1.9), these variables were found to be independently associated with the treatment outcome even after adjustment using multivariate analysis.

**Table 4:** Risk measurement among older TB patients registered under RNTCP in 12 districts of Tamilnadu, South India based on the Treatment Outcome (N = 1481), April to June 2011.

Category	Subcategory	Unfavourable Outcome^a^ N(%)	Favourable Outcome^b^ N(%)	Unadjusted RR^c^ (95% CI)	Adjusted RR (95% CI)
**Age groups**	70 years and more	78 (22)	277 (78)	1.6 (1.3–2.0)	**1.5** (**1.2–1.9**)
	60 to 70 years	155 (14)	971 (86)	Ref	
**Sex**	Male	201 (17)	988 (83)	1.5 (1.1–2.2)	**1.5** (**1.0–2.1**)
	Female	32 (11)	260 (89)	Ref	
**Category of TB**	New	178 (13)	1144 (87)	Ref	
	Retreatment	55 (35)	104 (65)	2.6 (1.2–3.3)	**2.5** (**1.9–3.2**)
**Site of TB**	Pulmonary	212 (16)	1091 (84)	1.4 (0.9–2.1)	Not Included
	Extra pulmonary	21 (12)	157 (88)	Ref	
**HIV status** *	Positive	5 (22)	18 (78)	0.9 (0.8–1.2)	1.3 (0.7–2.7)
	Negative	225 (15)	1230 (5)	Ref	
**DOT** d	Community-based	40 (21)	153 (80)	1.4 (1.0–1.9)	**1.4** (**1.1–1.9**)
	Healthcare based	193 (15)	1095 (85)	Ref	

a Favourable Outcome  =  Cured and Treatment Completed.

b Unfavourable Outcome  =  Died, Loss to follow up, Failure, Transferred out.

c Relative risk.

d Direct Observed Treatment.

* 3 Unknown HIV status was omitted.

TB – Tuberculosis.

RNTCP- Revised National TB Control Program.

## Discussion

The present study has highlighted the profile and treatment outcomes of the older TB patients in Tamilnadu, South India. Older TB patients accounted for 14% of all TB patients and had 38% higher risk of unfavourable treatment outcome as compared to all other TB patients. These rates are comparable to the study done in West Bengal during 1999–2005 and are higher as compared to study done in Delhi (1999–2001). In contrast to previous studies on older TB patients done in tertiary heath care settings or small geographical areas, this study included a large cohort of TB patients from 12 districts of Tamilnadu and is more likely to be representative of programme performance. The 12 districts are representative of state of Tamilnadu. In terms of geographical coverage these districts are distributed across the state without any particular skew-ness in any direction and have comparable indicators with respect to district TB performance and other health indicators [2], [18].

The findings of this study have many important implications for the national programme. The 4: 1 ratio of male to female older TB patients follows the epidemiological pattern of TB in this geographical setting from results of the prevalence surveys conducted in Tamil Nadu [20], [21] and allays fears of under-notification among females. The case notification among older TB patients was calculated to be 259 per 100,000 population as compared to other TB patients where it was found to be 142 per 100,000 population which again follows epidemiological patterns previously observed in Tamilnadu [20], [21].

The treatment outcomes among older TB patients were poor with higher death and loss to follow-up rates, a finding confirmed by previous studies in India and Africa between 1999 and 2010 [3]–[7].

Death rates were more than those routinely reported for all TB patients (6%) in the state of Tamilnadu. Among older TB patients, we found that unfavourable treatment outcomes were more likely in those aged 70 years and above, males, retreatment TB patients and those who received the community-based DOT. This calls for increased attention to this sub-group of TB patients by the national TB programme. The higher mortality is particularly worrying and may be related to the high levels of co-morbid conditions like diabetes mellitus, hypertension and cardiovascular diseases in these patients. Since this study was a record review of TB registers with lack of recording on co-morbidities, we could not assess the exact extent of this problem. Given the high prevalence of diabetes mellitus among TB patients in South India including Tamilnadu, it is important to routinely test every TB patient, especially those in the older age group for Diabetes mellitus and link them to appropriate diabetes care services [22], [23]. It is also necessary to link them with the National Programme for prevention and control of Cancer, Diabetes mellitus, cardiovascular diseases and stroke (NPCDCS) [24] for assessment of all co-morbidities. This calls for a close collaboration between RNTCP and NPCDCS under the overall umbrella of National Rural Health Mission of Government of India. This collaboration should give particular attention to making appropriate modifications in the existing recording and reporting system and consequent enhanced monitoring. With the introduction of a web-based case-based recording system under RNTCP, this would enable periodic monitoring of older TB patients.

HIV infection was associated with higher mortality, though not found to be statistically significant in multivariate analysis, possibly due to low numbers. Though assessment of HIV status was done in nearly 99% of TB patients, nearly 40% of HIV-infected TB patients did not receive ART and CPT. This finding calls for immediate attention, given the maturity of TB/HIV collaborative activities in the state.

The Revised National TB control program offers for all the registered TB patients the different options for DOT. Based on the location, timing and convenience, the patients opt either for DOT with the health provider or with a community volunteer. Community-based DOT is being promoted with provision of incentives by the national programme in an effort to increase the reach of the services and make them patient friendly. However, this study found out that older TB patients who received their treatment by a community based volunteer/health care worker had a higher loss to follow-up. Though, the exact reason for this is not known, it may be due to lack of ability of community providers to manage adverse reactions which are quite likely to be common in this age group or difficulty for the patients to walk to their DOT providers considering their age. It is also important to enhance the supervision and monitoring of such decentralized services, assess exact reasons and take appropriate remedial actions.

The issue of poorer treatment outcomes among males and retreatment TB patients is well known among all TB patients and is not specific to older age group.

The study had a few limitations. Since the study was a review of routinely maintained records, it was not possible to validate the data. However the recording and reporting system in RNTCP is quite robust and is monitored for data quality by dedicated and trained staff. As mentioned earlier, information about the presence of co-morbidities was not recorded in the TB register and hence we could not assess or adjust for this important confounder. We chose the study districts and study period purposively based on the area of work or principal investigator and not in a random manner raising concerns about the generalizability of results. However, the 12 districts are geographically spread across the state and are similar to the other districts in the state with respect to many socio-economic and health related indicators [2], [18]. Hence, we believe that the findings may be generalizable. Since the treatment outcomes do not show any seasonal variation, the findings coming from TB patients registered in one quarter of the year are unlikely to be any different if we studied the entire year.

In conclusion, we found that treatment outcomes were poor in older TB patients warranting special attention to this group – including routine assessment and recording of co-morbidities, a dedicated recording, reporting and monitoring of outcomes for this age-group and collaborate with collaborate with National programme of non-communicable diseases for comprehensive management of co-morbidities.
